# A Bayesian maximum entropy model for predicting tsetse ecological distributions

**DOI:** 10.1186/s12942-023-00349-0

**Published:** 2023-11-16

**Authors:** Lani Fox, Brad G. Peter, April N. Frake, Joseph P. Messina

**Affiliations:** 1 Lani Fox Geostatistical Consulting, Claremont, CA USA; 2 Department of Environmental Sciences and Engineering, Gillings School of Public Health, University of North Carolina at Chapel Hill, Chapel Hill, NC USA; 3 Department of Geosciences, University of Arkansas, Fayetteville, AR USA; 4 Department of Geography, University of Alabama, Tuscaloosa, AL USA; 5 Center for Global Change and Earth Observation, Michigan State University, East Lansing, MI USA; 6 Center for Healthy Communities, Michigan Public Health Institute, Okemos, MI USA

**Keywords:** Tsetse, Mitigation, Kriging, Bayesian maximum entropy, Google Earth Engine, Geospatial modeling

## Abstract

**Background:**

African trypanosomiasis is a tsetse-borne parasitic infection that affects humans, wildlife, and domesticated animals. Tsetse flies are endemic to much of Sub-Saharan Africa and a spatial and temporal understanding of tsetse habitat can aid surveillance and support disease risk management. Problematically, current fine spatial resolution remote sensing data are delivered with a temporal lag and are relatively coarse temporal resolution (e.g., 16 days), which results in disease control models often targeting incorrect places. The goal of this study was to devise a heuristic for identifying tsetse habitat (at a fine spatial resolution) into the future and in the temporal gaps where remote sensing and proximal data fail to supply information.

**Methods:**

This paper introduces a generalizable and scalable open-access version of the tsetse ecological distribution (TED) model used to predict tsetse distributions across space and time, and contributes a geospatial Bayesian Maximum Entropy (BME) prediction model trained by TED output data to forecast where, herein the *Morsitans* group of tsetse, persist in Kenya, a method that mitigates the temporal lag problem. This model facilitates identification of tsetse habitat and provides critical information to control tsetse, mitigate the impact of trypanosomiasis on vulnerable human and animal populations, and guide disease minimization in places with ephemeral tsetse. Moreover, this BME analysis is one of the first to utilize cluster and parallel computing along with a Monte Carlo analysis to optimize BME computations. This allows for the analysis of an exceptionally large dataset (over 2 billion data points) at a finer resolution and larger spatiotemporal scale than what had previously been possible.

**Results:**

Under the most conservative assessment for Kenya, the BME kriging analysis showed an overall prediction accuracy of 74.8% (limited to the maximum suitability extent). In predicting tsetse distribution outcomes for the entire country the BME kriging analysis was 97% accurate in its forecasts.

**Conclusions:**

This work offers a solution to the persistent temporal data gap in accurate and spatially precise rainfall predictions and the delayed processing of remotely sensed data collectively in the − 45 days past to + 180 days future temporal window. As is shown here, the BME model is a reliable alternative for forecasting future tsetse distributions to allow preplanning for tsetse control. Furthermore, this model provides guidance on disease control that would otherwise not be available. These ‘big data’ BME methods are particularly useful for large domain studies. Considering that past BME studies required reduction of the spatiotemporal grid to facilitate analysis. Both the GEE-TED and the BME libraries have been made open source to enable reproducibility and offer continual updates into the future as new remotely sensed data become available.

## Background

### Tsetse and African trypanosomiasis

Trypanosomiasis is a debilitating and potentially fatal disease to humans and domestic animals presenting significant health and economic challenges across 37 countries of sub-Saharan Africa [1]. Trypanosomes transmitted by the bite of the tsetse fly (genus *Glossina*) [2] cause African Animal Trypanosomiasis (AAT), also known as Nagana, and Human African Trypanosomiasis (HAT), also known as sleeping sickness. Tsetse flies are K-strategists, with long life expectancy (average of 90 days per female) and high survival rates (> 90% daily survivorship in adults), combined with low reproduction rates of one live pupa deposited in a suitable soil every 6 to 9 days [3]. Both animals and humans contribute to *Trypanosoma* genetic exchange [4, 5]. The tsetse fly carries the parasites to different animal hosts, allowing cyclical transmission, but the primary animal reservoirs are wild ungulates and domestic cattle. Methods to control the tsetse fly include the use of traps, targets [6–8], and less commonly pyrethroid-treated cattle [9].

In Kenya, eight species of tsetse fly occupy diverse habitats in spatially discontiguous “fly belts” that reflect common climatological, edaphic, and landscape characteristics disjoint by unsuitable habitat [10, 11]. While tsetse flies are classified into a single genus, *Glossina*, there are three extant subgenera organized according to species physiology and habitat preferences: *Morsitans*, *Palpalis*, and *Fusca* which are generally restricted to savannah, riverine, and forested habitats, respectively. It is estimated that 38 of 47 counties in Kenya are affected by tsetse flies [12], with the *Morsitans* group being the most widely distributed across the country [36]. Multiple tsetse fly species can coexist in the same area, sometimes making it difficult to quickly identify any single causative agent in human or animal epidemics [5], and the within belt spatial and temporal distributions can vary significantly [13, 14].

There are three major *Trypanosoma* species that cause AAT in cattle throughout Kenya: *Trypanosoma congolense* (subgenus *Nannomonas*), *T. vivax* (subgenus *Duttonella*), and *T. brucei* subspecies *brucei* (subgenus *Trypanozoon*), and *T. simiae* [15]; human infection of AAT is rare [16]. AAT is associated with substantial economic losses, primarily by constraining the livestock industry [1, 15–20] through high costs of veterinary care, lower calving rates and milk yields, and higher rates of calf mortality [21]. Economic losses from AAT to cattle production across sub-Saharan Africa are estimated at US$ 1.0–1.2 billion; annual agricultural Gross Domestic Product (GDP) losses are US$4.75 billion [22]. Losses from AAT in Kenya are estimated at US$ 200 million [12] and disproportionately affect impoverished livestock farmers. While notable progress has been made to combat AAT infection, it continues to be a major obstacle to improved livestock productivity throughout sub-Saharan Africa [15, 23].

There are two distinct parasites that cause HAT: *Trypanosoma brucei gambiense* (gHAT) is responsible for the Gambian form of the disease in 24 countries in Central and West Africa, and *T. b. rhodesiense* (rHAT) is responsible for the Rhodesian form found in 13 countries in East and Southern Africa. The gHAT form is responsible for 98% of infections [24]. Beyond direct health outcomes, HAT has considerable socio-economic impacts including declines in agricultural productivity from disruption to daily activity, food insecurity both from reduction of agricultural productivity and availability of meat and milk, disruption of children’s education from missed school days [25], and hindered agricultural development and prosperity [26]. Until 2021, treatment options for HAT required infusion, injection, or hospitalization which presented significant challenges for patients living in remote areas [27]. Fexinidazole, the first all-oral treatment for both stages of HAT, was approved by the U.S. Food & Drug Administration in July 2021 for patients 6 years and older that weigh ≥ 20 kg [28]. There are no prophylaxis or vaccines currently available for either form of HAT, which elevates the importance of control measures.

### Identifying tsetse presence and targeting disease management

The World Health Organization (WHO) targeted the elimination of HAT as a public health problem by 2020 in its 2012 Neglected Tropical Diseases (NTD) roadmap [29]. Since then, progress toward HAT elimination has involved myriad stakeholders and led to a reduction of gHAT cases by 96% and rHAT cases by 97% from 2009 to 2018 [30]. By 2030, disease endemic countries and the WHO aim to eliminate transmission of gHAT; the zoonotic nature and more complicated pathology of rHAT makes elimination far more complicated and not currently considered feasible [30–32].

Climate change will have complex impacts on tsetse fly, AAT, and HAT distributions. Tsetse fly population dynamics are inextricably linked to environmental drivers such as temperature and vegetation structure that constrain spatial distribution and potential density. Continued progress toward AAT and HAT elimination in the wake of climate and land cover changes must account for spatial and temporal expansion rates of tsetse species, following favorable changes in local or regional weather, and longer term land-cover changes such as greening or bush expansion in areas resulting from increased rainfall over multiple years [33]. This study builds on previous work that captured Tsetse dynamics 45 days after remote sensing data collection and a predictive infectious disease agent based model [14, 34]. Here, we seek to expand our modeling portfolio with an agile modeling methodology that is customizable by tsetse species or group to better capture and thereby reduce the critical time period between remote sensing data collection and tsetse control activities in the field. For the purposes of this study the model is parameterized for the *Morsitans* group within the tsetse fly genus *Glossina*. The second major aim of this paper was to advance the modeling environment developed in DeVisser et al. [35] to one widely available, easily updatable, and low/no cost to affected countries.

### Fundamental niche vs. realized niche and the time lag problem

The literature on predicted disease vector distributions is robust where the aim of different models is to predict species’ fundamental or realized niche [36–40]. Fundamental niche is the geographic space where environmental conditions allow the modeled species to exist, absent biotic interactions. Realized niche considers biotic interactions including competitive exclusion and is the space a species is known to occupy [41–43]. The outcomes of this model are intended to identify fundamental niche and plausible habitat in Kenya by elucidating geographical spaces at given times wherein environmental conditions and spatial connectivity permit the species to exist. In this paper, we assume tsetse populations have not been systematically cleared and species corridor blocking is not occurring, thus the fundamental and realized niches overlap.

**Fig. 1:**
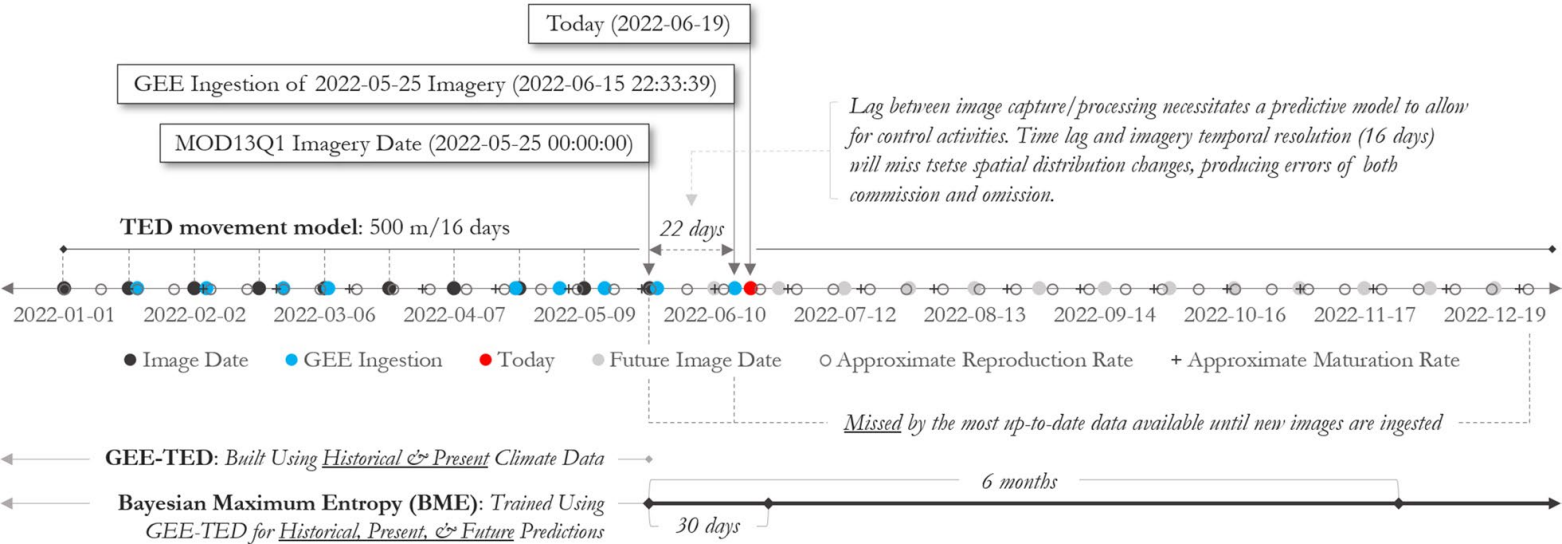
Timeline of MOD13Q1 imagery capture and GEE ingestion for use in GEE-TED. See “Methods” section for details on GEE-TED. Also depicted are future imagery dates, approximate reproduction rate, and approximate maturation rate. Today (the date of diagram creation) is included as a reference to show the lag between desired model run date and data availability. Approximate maturation and reproduction rates (16 and 9 days, respectively) are from Gooding and Krafsur [45]. The tsetse movement rate depicted here corresponds with the TED model by DeVisser et al. [35]

This paper addresses a critical temporal problem in the use of satellite imagery to model ‘current’ tsetse habitat, which is dependent on the revisit rate of the satellites, image data delivery, and the processing architecture used. There is inevitably some time lag between imagery capture and model output. In the case of GEE-TED [a Tsetse Ecological Distribution (TED) model that uses Google Earth Engine (GEE); refer “Methods” section for a complete description of GEE-TED], there must be imagery capture and subsequent ingestion into GEE before modeling can be performed; this can take multiple weeks (a 22 day lag is depicted in Fig. 1), though lag time varies per image. Once the MODIS biophysical data (i.e., temperature and NDVI) are ingested into GEE, the GEE-TED algorithm can be completed in minutes. The delay between MODIS image capture and data ingestion into GEE is particularly problematic during the wet season when tsetse movement is known to increase due to elevated humidity and decreased temperature [44]. Within the data gap time period, tsetse will have matured and may have reproduced multiple times, and would have been able to travel at least 500 m (Fig. 1) [35, 45]. From April 7th, 2003 to May 9th, 2003—a 32 day timespan—the habitable area for tsetse (as measured via GEE-TED) increased by ~ 12.8%, an overall change in area of approximately 5719 square kilometers. If tsetse habitat is modeled at the later stages of a data gap during the wet season, large swaths in need of tsetse remediation could be completely overlooked.

The heuristic presented here addresses the time lag problem by introducing a Bayesian Maximum Entropy (BME) kriging model that uses GEE-TED as a training tool to predict tsetse habitat during the data gaps, as well as further into the future, to more accurately convey ‘current’ tsetse habitat at more points in time. Outputs from this analysis and studies utilizing this heuristic will provide needed information to target both the where and when of tsetse habitat for more effective disease relief and prevention. Furthermore, GEE-TED is openly accessible and designed such that results from this study can be replicated for Kenya, as well as new maps generated for any region so long as site-specific tsetse species parameterizations are known. Likewise, the BME kriging model used here is also openly accessible and generalizable across geographies.

One of the major strengths of our approach is utilizing cluster and parallel computing in conjunction with a Monte Carlo model for the BME analysis. A past limitation of BME methodology is the inability to process large datasets using fine spatiotemporal scales because of the computationally intensive nature of the calculations. Current literature has concluded parallel computing could be utilized to maximize BME’s computational efficiency [46–49]. To the best of our knowledge, our proposed framework is amongst the first to implement parallel computing in conjunction with a Monte Carlo analysis to optimize the BME methodology.

## Methods

To predict *Morsitan* group tsetse presence in Kenya the existing Tsetse Ecological Distribution (TED) model—a dynamic raster species distribution model was used [35]. As described in DeVisser et al. [35], TED integrates four Moderate Resolution Imaging Spectroradiometer (MODIS) datasets: (i) Normalized Difference Vegetation Index (NDVI, MOD13Q1) [50], a proxy for soil moisture, (ii) day land surface temperatures (LST, MOD11A2) [51], (iii) night LST, and (iv) Land-Use/Land-Cover (LULC, MCD12Q1) [52, 53] to create a location-based fundamental niche model of *Morsitan* group tsetse presence [35]. TED rational, variables, and model creation are described in detail in [35]. TED generates a post hoc map of tsetse suitability every 16 days (the temporal resolution of MOD13Q1) at a 250-m spatial resolution using the MODIS datasets to assess binary habitat suitability. The suitability maps are then aggregated utilizing Boolean logic to populate a fundamental niche map computing presence probability [35]. In addition to habitat suitability, TED integrates tsetse movement rates to account for seasonal expansion and contraction of the geographic niche [35].

**Fig. 2:**
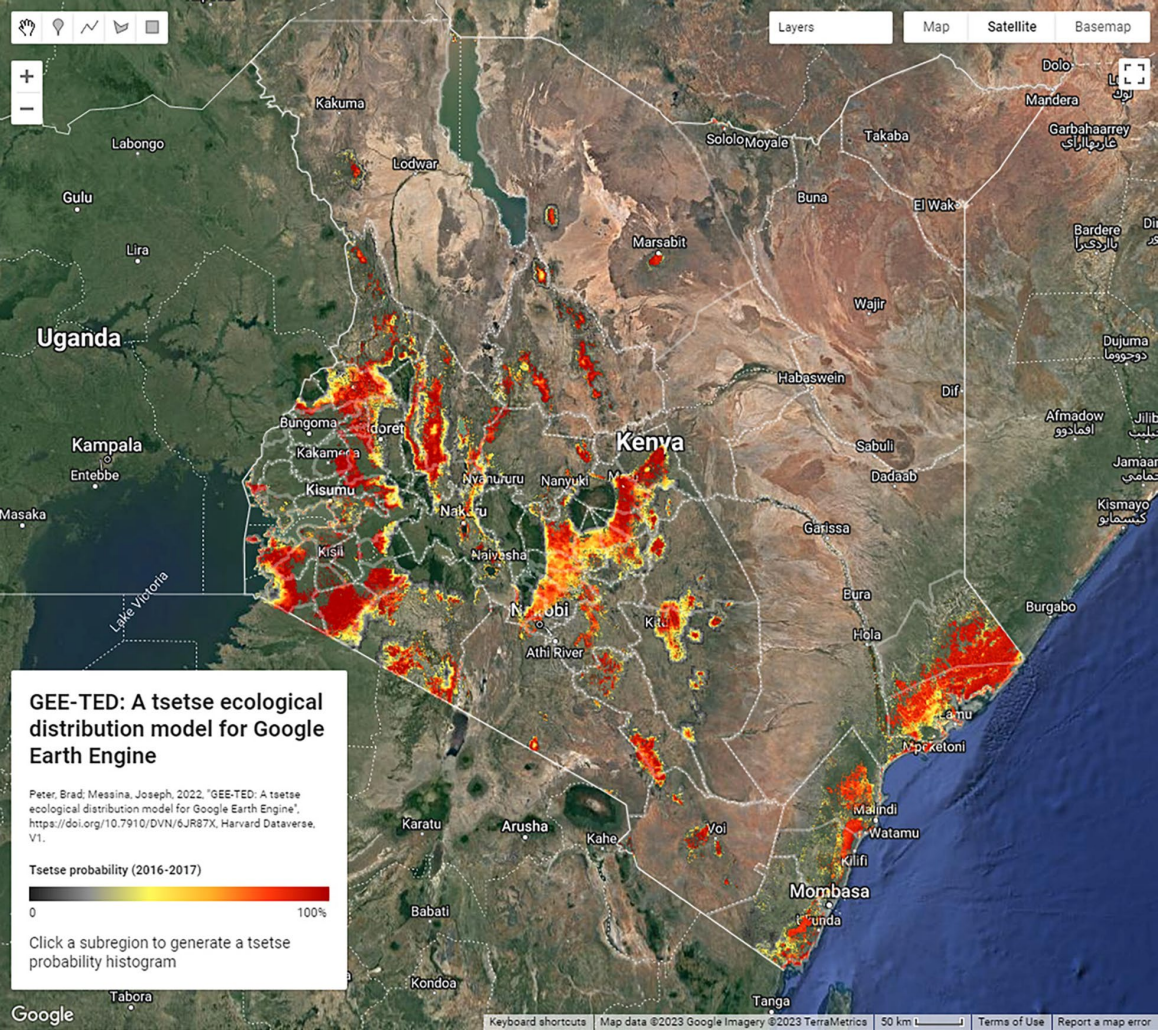
Sample GEE-TED output showing the spatial variability of *Morsitan* group tsetse for 2016–2017 alongside Google satellite imagery. An interactive version of this map is available at https://cartoscience.users.earthengine.app/view/gee-ted and the code is accessible via [56]

The TED model used in this research was adapted from the original ArcPy heuristic developed by Devisser et al. [35] to function in Google Earth Engine (GEE) using the JavaScript API [54], henceforth referred to as GEE-TED. All data products used in the original TED model (MOD13Q1, MOD11A2, and MCD12Q1) are available in GEE. This adaptation was constructed to leverage GEE’s infrastructure for acquiring and processing spatially continuous and up-to-date MODIS climate and LULC products. GEE-TED performs the same as the original TED model, with one exception; in the adapted GEE-TED, one LULC layer was generated to mask suitability rather than masking based on yearly LULC. The mode of both versions of the MCD12Q1 product (V006 and V051) was calculated and a binary suitability grid produced where either or both versions showed a suitable LULC type. The decision to treat LULC in this manner was based on documented uncertainty associated with pixel ‘flipping’ (i.e., land classifications alternating due to subtle thresholds) occurring in the MCD12Q1 products [55]. GEE-TED is openly accessible via Harvard Dataverse [56]. Figure 2 shows a sample GEE-TED output alongside a satellite view of Kenya.

**Fig. 3:**
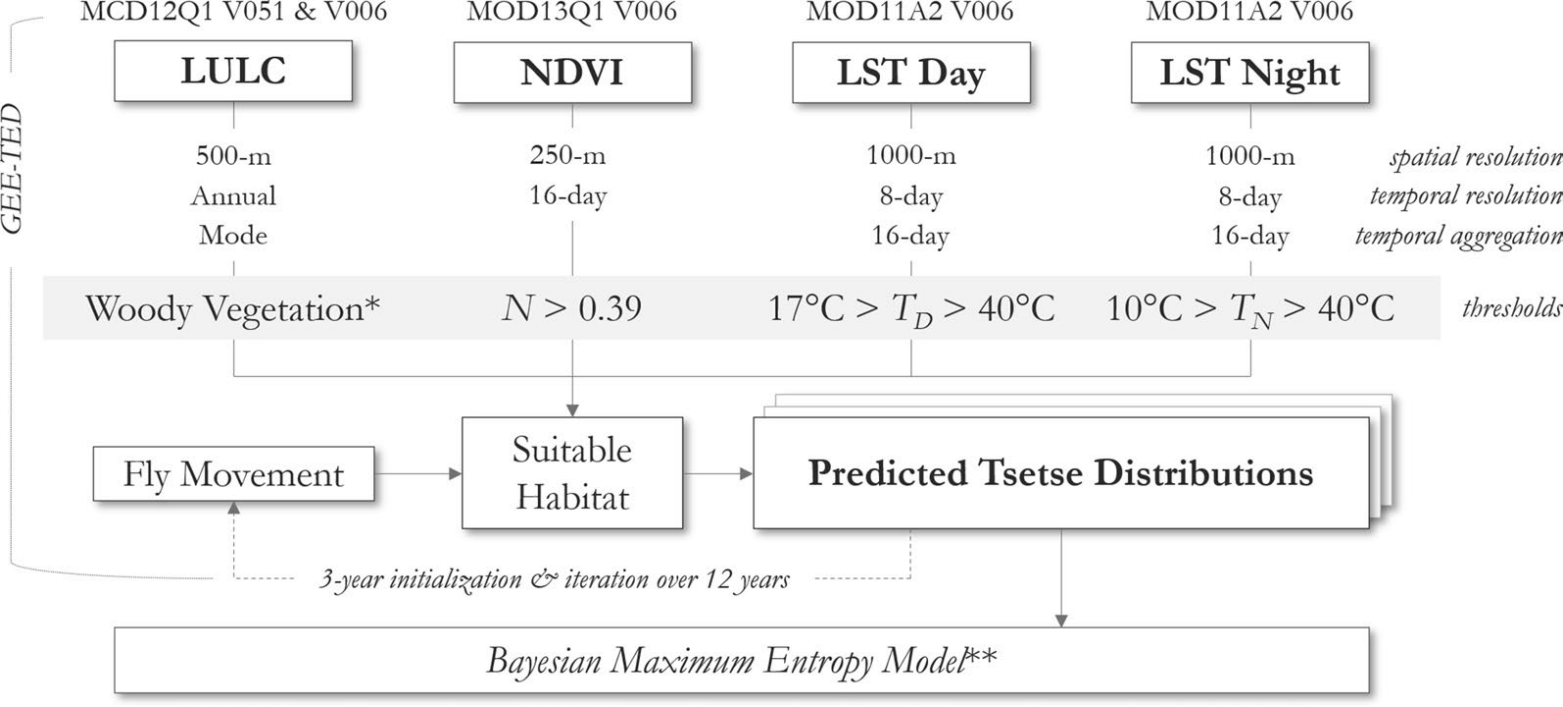
GEE-TED model diagram. *Suitable LULC types from DeVisser and Messina [43]. **Outputs from GEE-TED were used in the BME model

Suitable LULC classes (e.g., woody vegetation) were selected using the parameters recommended by DeVisser and Messina [57]. Following the original TED parameterization for *Morsitans* group tsetse flies*,* climate and land suitability thresholds included: NDVI > 0.39, day LST between 17 and 40 °C, and night LST between 10 and 40 °C [35]. Years under observation for this analysis were 2006 through 2017, with a 3-year model initialization from 2003 to 2005 (Fig. 3). The results from this model were used to train a BME kriging model. While this manuscript focuses on the *Morsitans* group of tsetse flies, the model may be re-parameterized to support identifying the spatial distribution of other tsetse species or groups as the code for GEE-TED is fully open access [56]; refer “Discussion and conclusions” section for additional commentary.

### BME analysis

BME is a space–time geostatistical kriging method using space/time random field (S/TRF) theory to minimize mean squared error (MSE) and provide the Best Linear Unbiased Predictor (BLUP) to estimate spatiotemporal random fields. In S/TRF theory, variables such as tsetse presence are given a value at a space/time coordinate (latitude, longitude, and time period). The S/TRF is built upon this known data and is itself a collection of possible values to predict the unknown spatiotemporal distribution of a variable [58]. A BME kriging analysis has three primary procedures. First, the S/TRF’s prior probability density function (PDF) is estimated. A covariance function representing the S/TRF’s space–time variability is calculated and used to develop the prior PDF. Second, a posterior PDF is found using Bayesian conditionalization. Finally, the posterior PDF is used to obtain space–time estimates of tsetse presence in Kenya, represented as spatial random fields [59–65]. These estimates are then used to create maps of predicted *Morsitans* group tsetse presence across Kenya. Detailed information on BME’s mathematical framework has been thoroughly described [58, 66–69]. Additionally, BME code libraries are freely available along with BMEGUI, a Graphical User Interface option to fully automate the BME model creation [59].

Numerical processing of the BME kriging analysis was performed using the University of North Carolina at Chapel Hill’s Longleaf computing cluster (https://its.unc.edu/research-computing/longleaf-cluster/). Computational analysis was completed on the cluster utilizing MATLAB 2017a [70] harnessing the BMElib 2.0b package, a freely available MATLAB library [71] which can be downloaded at https://mserre.sph.unc.edu/BMElab_web/ and MATLAB’s parallel computing toolbox [72].

Twelve years of GEE-TED model results (2006–2017; 276 MODIS time periods at a 16-day temporal resolution; 9,319,435 pixels per image) were used to train the BME model to predict tsetse presence in Kenya. In the BME model, GEE-TED results from 2006 to 2015 were used as the training set and data from 2016 to 2017 were used to validate the BME predictions. The raw training dataset is massive, consisting of more than 2 billion unique data points. This dataset was too large to calculate the computationally intensive algorithms of the BME kriging methods. Thus Kenya was partitioned into five vertical segments (the minimum number of partitions so that the processing system could handle the data workload) and a 25 km buffer was added to each Area to minimize edge effects [73, 74]. The area partitioning was primarily for the purposes of data processing and aggregate results generated are indicative of the outcome had no partitioning taken place. However, results are presented at the country level and by areas since the area partitioning closely tracks the Kenyan ecoregions and elucidates differences across spatial domains.

To ensure the dataset meets the BME kriging requirements of a S/TRF that is homogeneous in space and stationary in time, the global mean trend is calculated and removed prior to the analysis. The global mean trend is a deterministic function in which the residual S/TRF models the uncertainties and variability associated with a dataset over space and time [50]. Removing the mean trend smooths spatiotemporal fluctuations and creates a dataset that is homogenous in space and stationary in time [60, 61, 67].

In a BME kriging model both a spatial and temporal mean trend are calculated and combined to create a global mean trend [60]. It is assumed the spatial component of the mean trend will remain relatively stable in the predicted years (2016 and 2017) and the spatial mean trend can be computed using the existing dataset. However, the temporal mean trend changes every time period, and we are predicting into the future (a modifiable temporal unit problem effect; [73, 74]), thus a trend based upon the average for each MODIS time period within the years 2006–2015 was used to estimate the temporal mean trend.

The mean trend-removed residual data are then used to conduct a covariance analysis for the dataset. Covariance is a measure of the association and direction of the linear relationship between two variables, describing how they change in relation to one another. A ‘covariance function’ is a quantitative description of the correlation between pairs of observations as a function of the inter-pair distances. The covariance model identifies how much weight/influence each neighboring point should exert in the calculation of a prediction [58, 60, 64]. Although the covariance model is a space–time function [64], the parameters for the temporal and spatial components are computed and investigated independently. Distinct space–time covariance models were created for each of the five Areas in Kenya based on a nested additive exponential space–time covariance model as shown in the following equation:

$${\text{c}}_{{\text{x}}} ({\text{r}},\tau ) = {\text{c}}_{1} \exp ( - 3{\text{r}}/{\text{a}}_{{{\text{r}}1}} )\exp ( - 3\tau /{\text{a}}_{{{\text{t}}1}} ) + {\text{c}}_{2} \exp \left( { - 3{\text{r}}/{\text{a}}_{{{\text{r}}2}} } \right)\exp ( - 3\tau /{\text{a}}_{{{\text{t}}2}} ) + {\text{c}}_{3} \exp \left( { - 3{\text{r}}/{\text{a}}_{{{\text{r}}3}} } \right)\exp ( - 3\tau /{\text{a}}_{{{\text{t}}3}} ).$$

To reduce run-time, the space and time covariance analyses were run in parallel for each Area, with 12 parallel ‘workers’ per Area. A worker is a computational driver that gives specific jobs to a computer’s computational sectors, often specific cores of a computer [72]. This method produced the temporal covariance. However, the spatial covariance analysis was too computationally intensive, maxing out the 1 TB memory available on the computing cluster. To solve this issue, a Monte Carlo analysis was used to estimate the spatial covariance for each of the Areas. To identify the optimum percentage of the dataset used and the number of Monte Carlo runs, two randomly selected testing locations with 50,000 continuous points were selected from each Area, totaling 10 test regions. The percentage of points used for each of the covariance Monte Carlo model tests was 1%, 2.5%, 5%, 10%, 20%, and 30%. The number of Monte Carlo trial runs was also tested using *N* = 10, 25, 50, 75, 100, and 250.

Once the results of the Monte Carlo trials were produced, two methods were used to evaluate the effectiveness of the prediction on the test regions and select the ideal parameters for the analysis. First, the test regions’ covariance, also known as the experimental covariance, was plotted to assess if there was any notable difference in the final covariance function that could be fitted to the test regions. Second, the average MSE was computed. Once the ideal conditions for increasing accuracy and decreasing run-time was found in the test regions, the spatial covariance analysis was run for each of the areas in Kenya.

After the covariance analysis was conducted, tests were run to identify the most appropriate kriging model for the dataset. For each of the five Areas in Kenya, two sets of 1000 spatially contiguous points with a randomly selected starting point were chosen. The kriging methods were then tested for four randomly selected time periods; two in 2016 and another two in 2017, resulting in 8 sets of predictions per Area. Five types of kriging models were tested: (i) simple kriging (no mean), (ii) ordinary kriging (constant mean), (iii) universal quadratic kriging (quadratic, constant + linear + quadratic mean), (iv) universal cubic kriging (cubic, constant + linear + quadratic + cubic mean) and (v) universal fourth power kriging (fourth power, constant + linear + quadratic + cubic + fourth power mean). As these are nested space–time models, 20 permutations of space–time kriging models were tested (e.g., simple kriging *spatial* model with an ordinary kriging *temporal* model). The MSE for the space and time kriging estimates was used to determine the model that was the most accurate predictor. The method with the lowest MSE for all the tests was then used to run the BME kriging model.

The final steps in the BME kriging analysis are to add the mean trend back to the residual data and create the final maps and statistics. Once the mean trend was returned to the data, the kriging results were rounded to either 1 or 0 (Boolean tsetse present or not present) to compare the results to the original GEE-TED model data. Additionally, the rounded results were used to make the final prediction maps and calculate MSE.

## Results

### Spatial and temporal mean trends

**Fig. 4:**
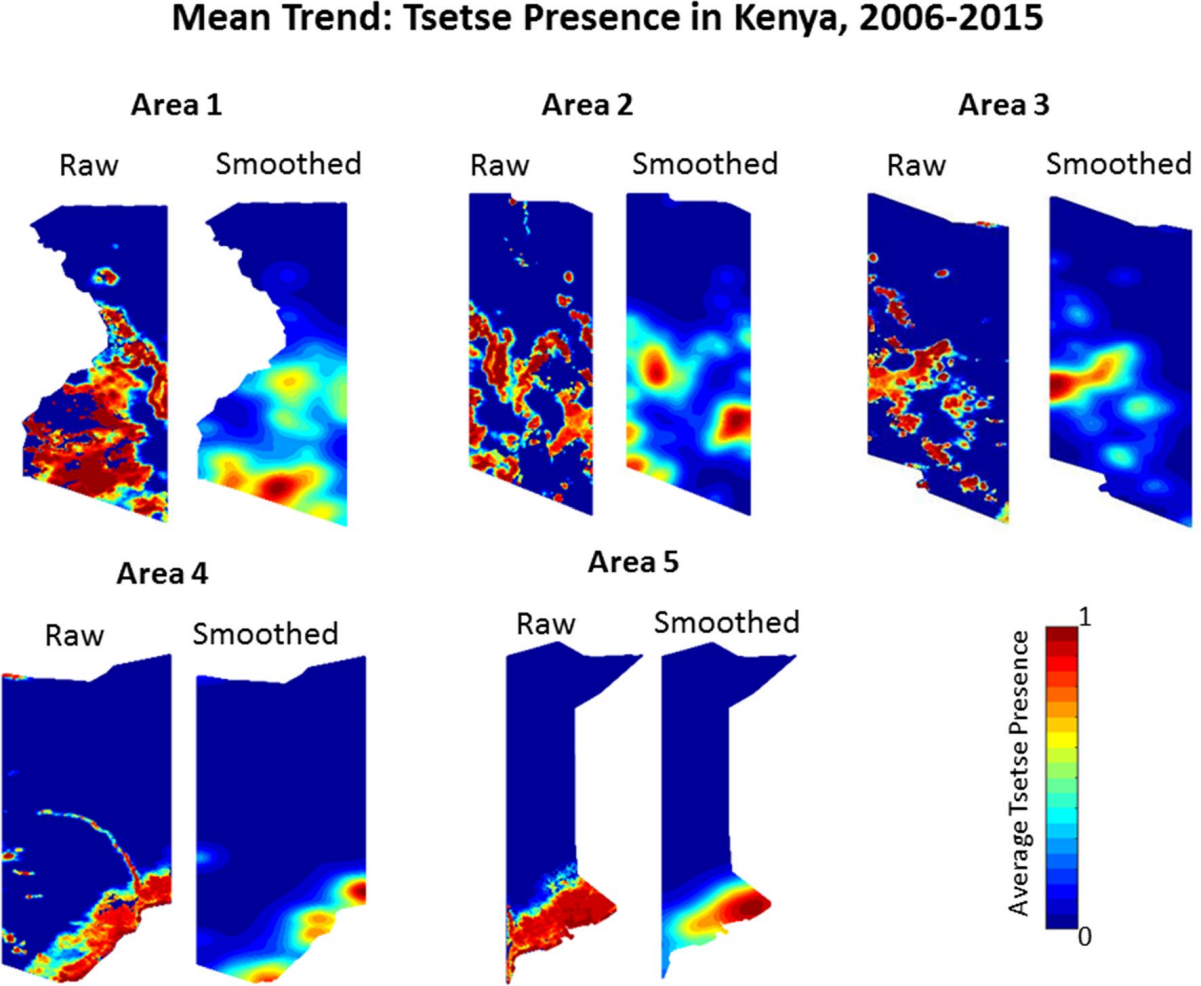
Spatial mean trend results for the five Areas in Kenya

The spatial and temporal mean trends were calculated for each Area in Kenya and are presented in Figs. 3 and 4. The spatial distribution of *Morsitans* group tsetse presence throughout Kenya is heterogeneous. Area 1 has the highest overall concentration of tsetse presence, primarily located in what is south-west Kenya along Lake Victoria and bordering Tanzania. In the other Areas, tsetse presence is most often in the central and southern regions. This is especially prevalent in Areas 4 and 5 where the spatial trend of tsetse presence is concentrated in the southern portion with little tsetse presence elsewhere. The arc in the raw Area 4 data extending from the west-central, to south-east is the River Tana.

**Fig. 5:**
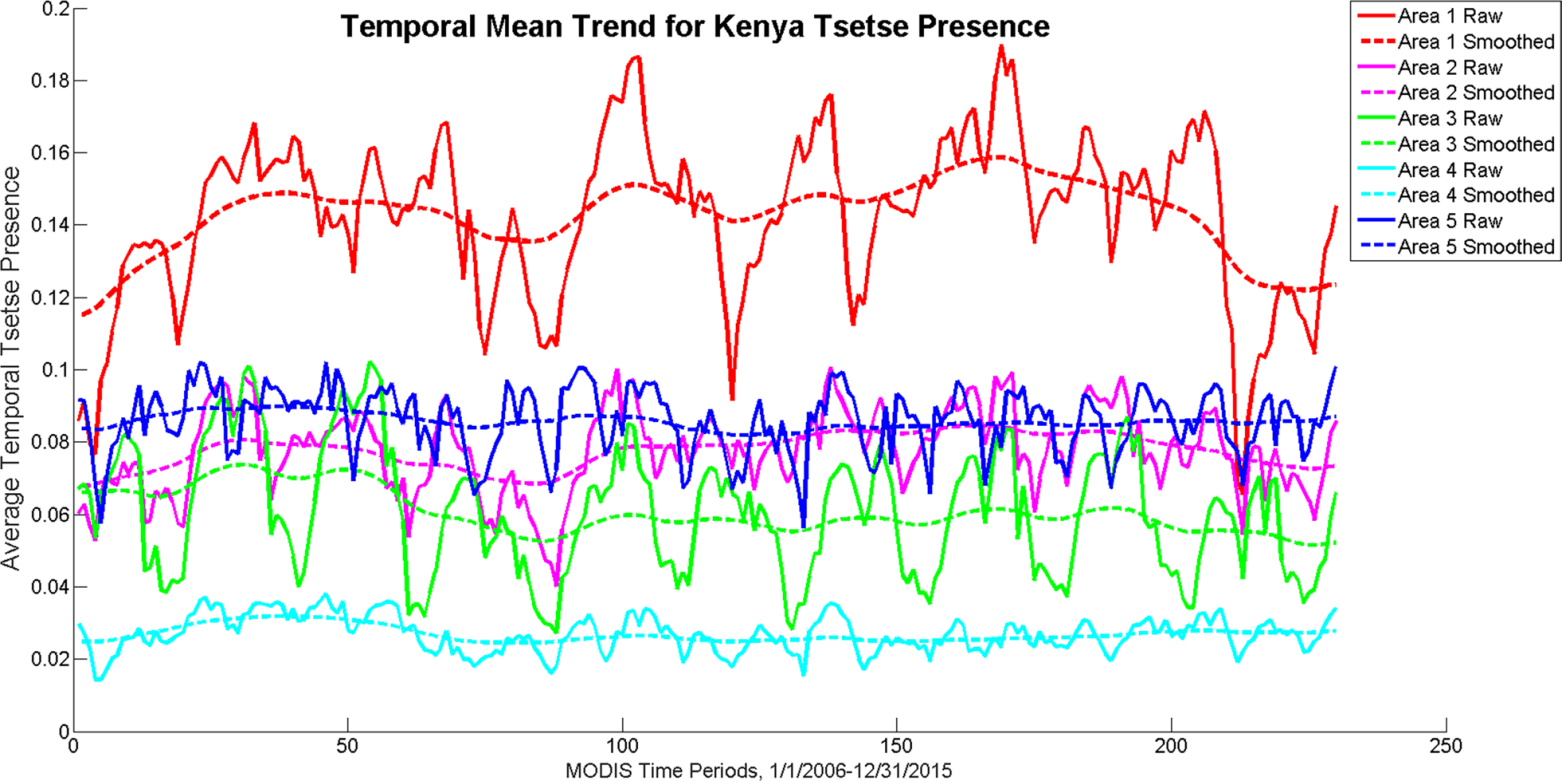
Temporal mean trend results for the five Areas in Kenya

In the temporal component (Fig. 5), all Areas have a cyclical pattern with increased tsetse presence in the rainy seasons and decreased tsetse presence in the dry seasons. Drought conditions can be seen in Area 1 in years 2006 and 2015 (MODIS time periods 2 and 210, which correspond with Jan. 17, 2006 and Feb. 2, 2015, respectively) and Area 2 in 2009 (MODIS time period 90, which corresponds with Nov. 17, 2009). Area 1 shows the greatest variation between the dry and wet seasons while Area 4 has the least variation between the dry and wet seasons.

### Monte Carlo results

To run the spatial covariance analysis, Monte Carlo simulations were performed. An optimization study was conducted to determine the most efficient and accurate methods for this analysis, i.e., the number of Monte Carlo trials and percentage of the dataset to be used. To select the ideal parameters for the Monte Carlo analysis, differences in resulting experimental covariance were produced using 30%, 20%, 10%, 5%, 2.5%, and 1% of the data, then plotted and visually assessed to ascertain any impact on a covariance model that would be fitted to the data. These tests found there were small differences in plots of the experimental covariance according to the percentage of spatial or temporal points used in the model; however, the differences were too small to impact the final fitted covariance model. Therefore, to optimize the efficiency of the analysis and reduce runtime, the best mean squared error (MSE) analysis was only performed on the datasets composed of 1%, 2.5%, and 5% of the total points. The MSE results for reducing both the percentage of spatial or temporal points in the model found 5% of the space and 5% of the time datasets produced the most accurate and efficient analysis (see Table 1). Finally, the number of Monte Carlo trial runs was investigated. Again, there were minimal differences in the covariance models that could be fitted to the data. The MSE results then showed that 50 trial runs minimized the run-time while preserving accuracy. Thus, the final parameters used in the spatial Monte Carlo covariance analysis were 5% of the dataset and 50 model runs.

**Table 1:** Monte Carlo analysis test results for spatial covariance

Spatial percentages			Temporal percentages			Runs		
Area and section	Best visual	Best MSE	Area and section	Best visual	Best MSE	Area and section	Best visual	Best MSE
1, 1	0.025	0.05	1, 1	0.025	0.025	1, 1	50	50
1, 2	0.05	0.05	1, 2	0.05	0.025	1, 2	10	50
2, 1	0.05	0.05	2, 1	0.05	0.05	2, 1	50	50
2, 2	0.05	0.05	2, 2	0.05	0.05	2, 2	50	50
3, 1	0.05	0.05	3, 1	0.05	0.05	3, 1	50	75
3, 2	0.05	0.025	3, 2	0.05	0.05	3, 2	50	75
4, 1	0.05	0.05	4, 1	0.05	0.05	4, 1	50	25
4, 2	0.05	0.025	4, 2	0.05	0.05	4, 2	50	50
5, 1	0.05	0.05	5, 1	0.025	0.025	5, 1	50	10
5, 2	0.05	0.025	5, 2	0.025	0.025	5, 2	50	10

### Final covariance models

The parameters for the final covariance model for each Area are shown in Table 2. The variable a_rx_ shows the spatial areas of influence for each component of the covariance; a_tx_ demonstrates the temporal areas of influence; c_x_ variable describes the portion of the covariance that part of the model represents.

**Table 2:** Covariance model parameters, where a_r_ is shown in m and a_t_ in MODIS time periods

Area	c_1_	a_r1_ (km)	a_t1_	c_2_	a_r2_ (km)	a_t2_	c_3_	a_r3_ (km)	a_t3_
1	0.045	2.5	2300, ~ 100 years	0.025	20	4, ~ 2 months	0.018	40	2300, 100 years
2	0.025	0.1	5000, ~ 217 years	0.02	27.5	6, ~ 3 months	0.009	22.5	5000, ~ 217 years
3	0.026	6	23,000, ~ 1000 years	0.008	2000	13, ~ 6 months	0.008	30	13, ~ 6 months
4	0.012	2	2300, ~ 100 years	0.005	17.5	5, ~ 2.5 months	[0.002, 5]	50	5, ~ 2.5 months
5	0.012	1	3, ~ 1.5 months	0.01	1	1000, 43.5 years	0.012	22.5	1000, 43.5 years

**Fig. 6:**
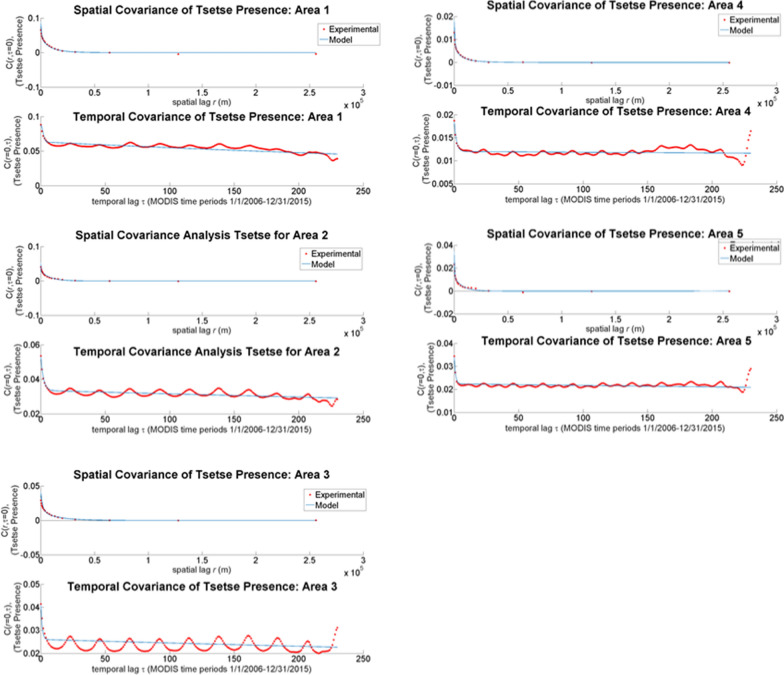
Covariance models for each of the areas in Kenya

Graphs of the covariance functions are shown in Fig. 6. By looking at the sill, also known as the covariance at distance 0, and the slope of the model near the sill, the spatial–temporal variability of the dataset is explained. The larger the value of the sill and the steeper the slope illustrates increased variability in the data [60]. All the Kenyan tsetse spatial covariance models show high spatial variability within the first 6 km. These temporal covariance models show great seasonal variation and a quick degradation of influence between points within 6 months, followed by seasonal stability in the dataset. The covariance range shows the temporal and spatial area that a data point will influence a neighboring estimation point in the model. The range is defined as the distance from the sill to the point where the model becomes asymptotic losing 95% of inter-pair correlation [60]. In this dataset, all the covariance models become asymptotic within 25 km and 10 MODIS time periods (~ 6 months), and thus points greater than 25 km and 6 months apart will exert minimal influence on an estimation. Therefore, demonstrating the 25 km overlapping border between the 5 Kenyan areas is sufficient to remove the edge effect. Because all these models have asymptotic curves, they show a dataset that is homogeneous in space and time [60, 64].

### Kriging results

Table 3 shows the total times each method performed best in the eight tests for the spatial component and the eight tests for the temporal component of kriging in each Area. In all Areas, simple kriging performed the best, producing the lowest MSE in most tests. Therefore, simple kriging was used for both the temporal and spatial components for the kriging analysis.

**Table 3:** Summarized Kriging type test results listing the number of times each method performed the best in each of the tests conducted

Key: [space, time, total]					
Area	Simple kriging	Ordinary kriging	Universal kriging: quadratic	Universal kriging: cubic	Universal kriging: fourth power
1	**5**, **3**, **8**	0, 0, 0	0, 0, 0	3, 3, 6	0, 2, 2
2	**5**, **2**, **7**	1, 0, 1	0, 3, 3	2, 1, 3	1, 1, 2
3	**2**, **7**, **9**	2, 0, 2	0, 1, 1	3, 0, 3	0, 1, 1
4	**7**, **5**, **12**	0, 0, 0	0, 3, 3	0, 0, 0	1, 0, 1
5	**4**, **7**, **11**	0, 0, 0	0, 1, 1	1, 0, 1	3, 0, 3
Total	**23**, **24**, **47**	3, 0, 3	0, 8, 8	9, 4, 13	5, 4, 9

### Geospatial accuracy

**Fig. 7:**
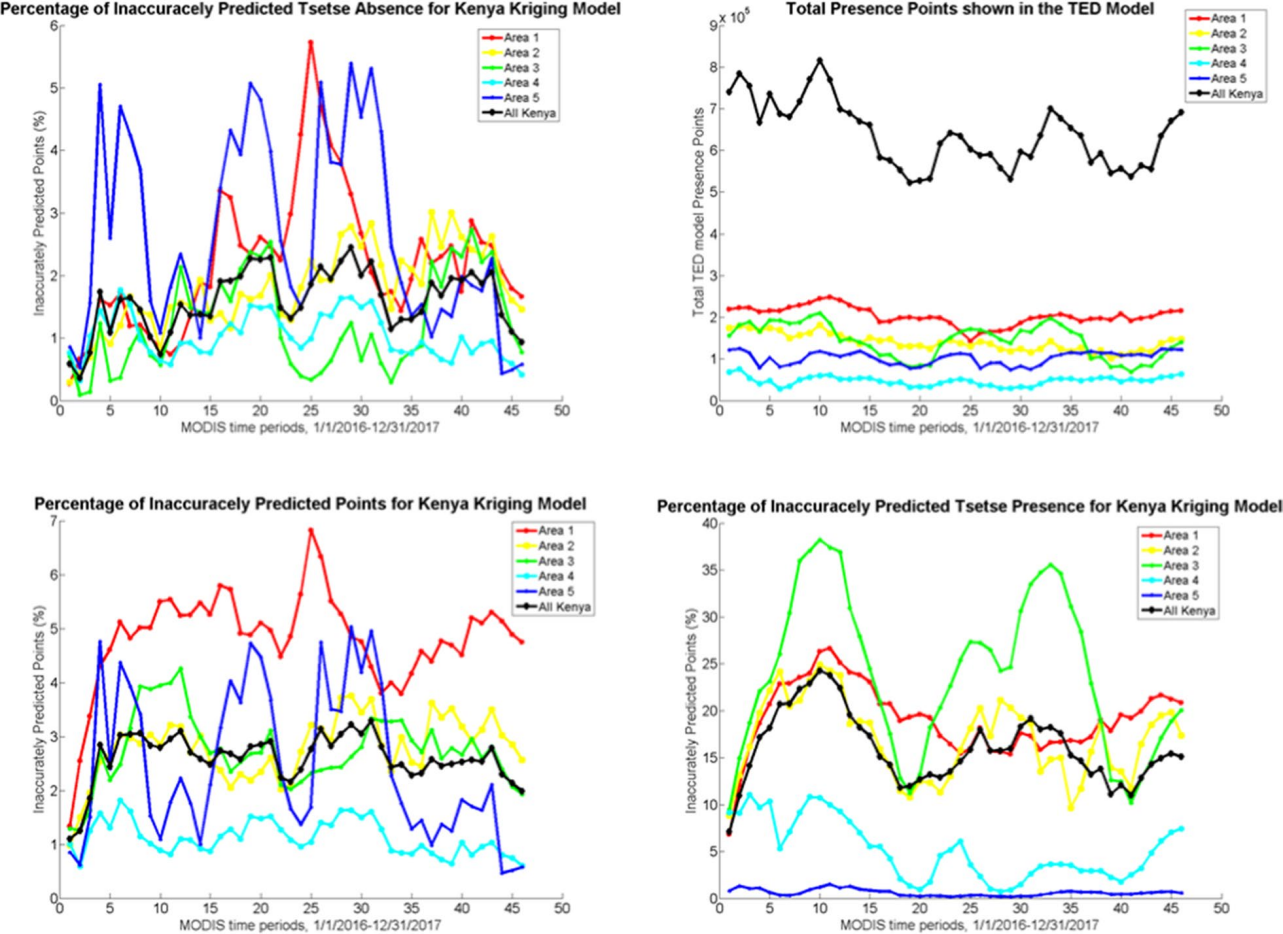
**a** All inaccurately predicted points (presence and absence) for the Kenya Kriging Model in 2016–2017, **b** presence data from the TED model over time (2016–2017) **c** inaccurately predicted absence from the BME model 2016–2017, **d** inaccurately predicted presence from the BME model 2016–2017

The kriging model forecasts accurately estimated the GEE-TED model outcomes more than 93% of the time when observed per area (Fig. 7); when the entire country is used, the total accuracy of the BME kriging prediction is 97–99%. Area 4 has the best predictions for the data followed by areas 5, 2 and 3. Area 1 has the most inaccurate predictions in Kenya (Fig. 7). As shown in Fig. 6, there is a slight decrease in the model’s ability to predict after the fifth MODIS time period, though the prediction stabilizes and does not have a subsequent decreasing trend in accuracy. However, accuracy does vary greatly within time periods due to seasonality. In Area 5, there is a cyclical nature to the prediction power of the kriging model that appears to be caused by seasonal variation. For Areas 2, 3, and 4 there are fluctuations throughout the time period; however, the ability for the model to predict based on all inaccurately predicted points stays relatively stable and possibly due to less seasonal variability in these regions. Total inaccurately predicted points are minimized the most in Area 4, and Area 1 exhibits the worst predictions.

The other notable result is the model is better at predicting absence than presence; there are many more absence points than presence, with total absence in the 10^6^ range (Fig. 7) and presence in the 10^5^ range (Fig. 7). Therefore, absence is dominant in the overall inaccuracy data. To clarify the use of the terms ‘inaccurate presence’ and ‘inaccurate absence’—an inaccurate presence prediction refers to the kriging model incorrectly classifying as absence where the GEE-TED model predicts presence, and an inaccurate absence prediction refers to the kriging model incorrectly classifying as presence where the GEE-TED model predicts absence. The prediction rate for absence is between 94 and 99.8% (Fig. 7). Areas 1 and 5 predicted absence the worst and Areas 4 and 3 predicted absence the best. The prediction accuracy for presence is between 65 and 99% (Fig. 7) with Area 3 having much larger issues with prediction than all of the other Areas; Area 5 has an almost perfect prediction for presence. For the other three Areas, the presence predictions are still high (between 75 and 90% accurate).

There are a few factors influencing the aggregated results for the entire country. First, the number of data points varies by Area. Area 4 has the largest number of points followed by Area 3, then 2, 1, and 5. There are two reasons for this. First Areas 2, 3 and 4 have buffers on both sides, whereas Areas 1 and 5 are on Kenya’s border and only have buffers on the interior sides. Second, the interior portions of the country have a larger y-axis and the x-axis was used to divide the country, therefore Areas 2, 3, and 4 have more data points. Consequently, the results of Areas 2, 3, and 4 have the greatest effect on the results for the country. Area 4 has the most absence points and Area 5 has the least, whereas Area 1 has the most presence points and Area 4 has the least. Area 4 having both the most absence, total points, and least presence gives it a strong effect on the country-wide results.

**Fig. 8:**
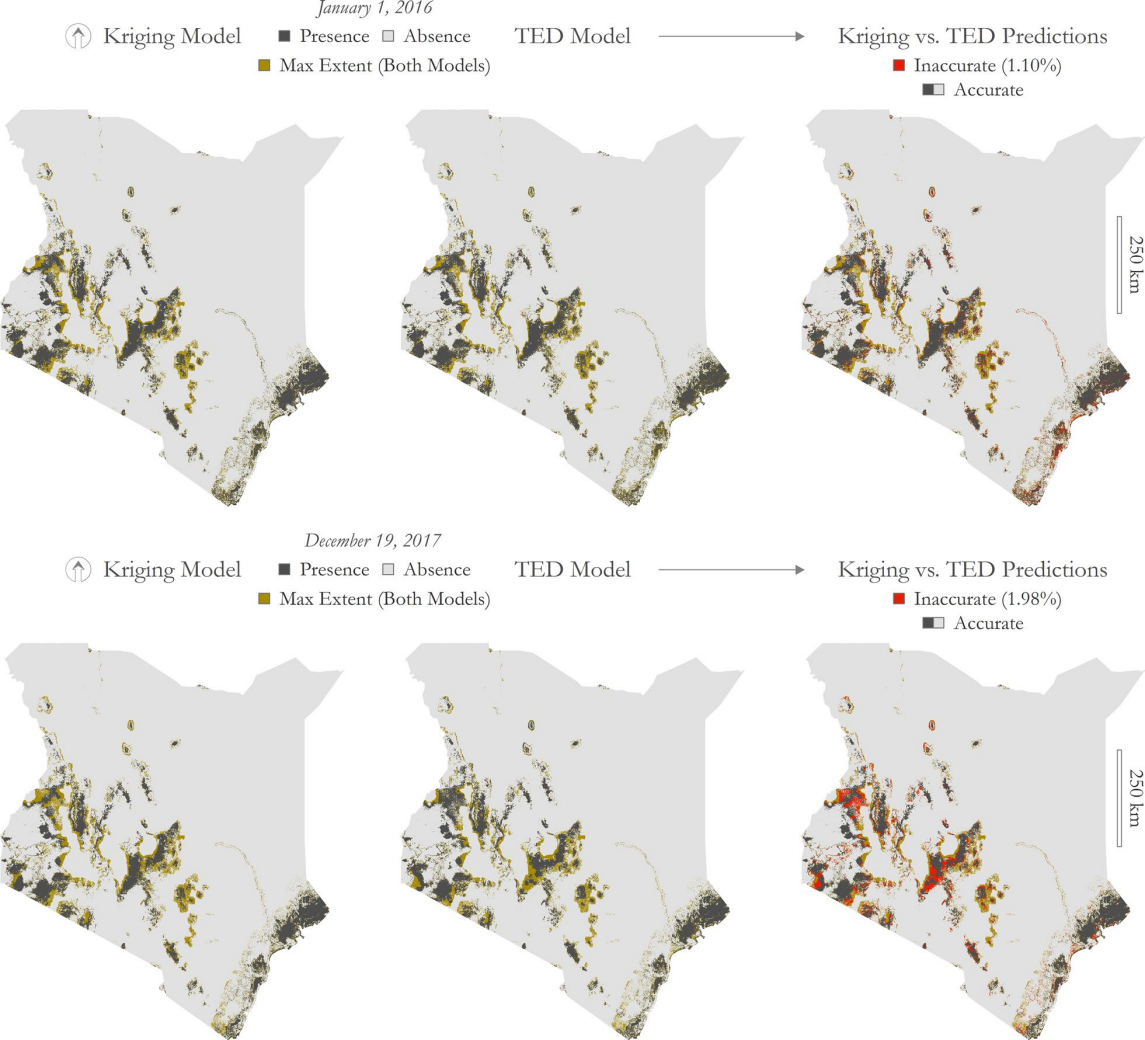
First and last kriging predictions compared to GEE-TED model results. Top: January 1, 2016; bottom: December 19, 2017. Top-left: kriging model results; top-middle: GEE-TED model results; top-right: comparison of results highlighting inaccurate predictions (depicted in red). Bottom-left: kriging model results; bottom-middle: GEE-TED model results; bottom-right: comparison of results highlighting inaccurate predictions (depicted in red). Presence predictions depicted in black and absence depicted in gray. Depicted in gold is the maximum extent of predicted presence across both models and across the complete temporal range under observation

Depictive maps are presented in Fig. 7 to illustrate the accuracy of the predictive kriging model at two specific time steps—the first and last days of the prediction range (January 1, 2016 and December 19, 2017). These two dates were selected because the kriging model stabilizes as it advances in time and these dates highlight a range of prediction accuracy (Fig. 8). On the first prediction date (January 1, 2016), only 1.10% of Kenya was inaccurately predicted (presence or absence), and on the last date (December 19, 2017), only 1.98% of Kenya was inaccurately predicted. Given that a large portion of Kenya is unsuitable for tsetse persistence (represented as tsetse absence on the maps), model accuracy appears very high; however, these metrics are susceptible to the modifiable areal unit problem [73–76], a term used to acknowledge that data aggregations can vary depending on the geographic extent under observation. To present a more conservative estimate of accuracy, the inaccurate predictions were also tabulated over only the maximum extent of the GEE-TED and kriging models (depicted in gold in Fig. 8). Under this geographic delineation, 9.95% of Kenya was predicted incorrectly on January 1, 2016 and 17.88% of Kenya was predicted incorrectly on December 19, 2017.

**Fig. 9:**
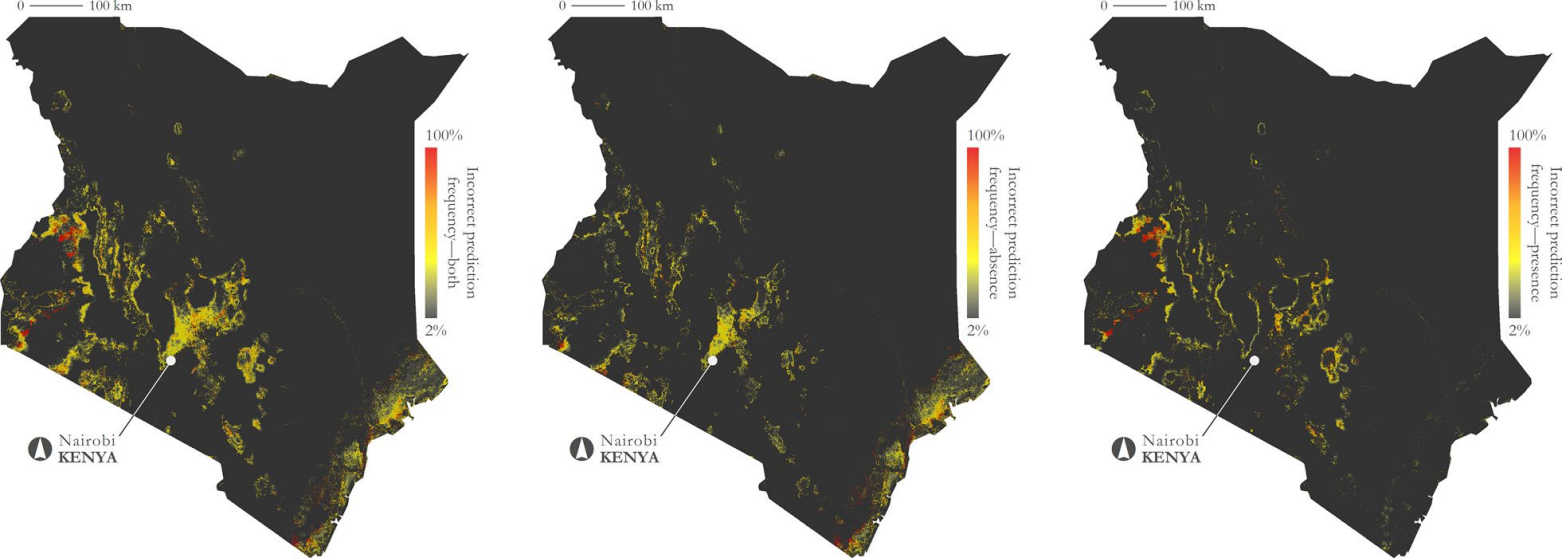
Frequency of inaccurate tsetse presence and/or absence predictions (2016–2017). Left: incorrect prediction frequency (absence and presence); middle: incorrect prediction frequency (absence); right: incorrect prediction frequency (presence)

**Fig. 10:**
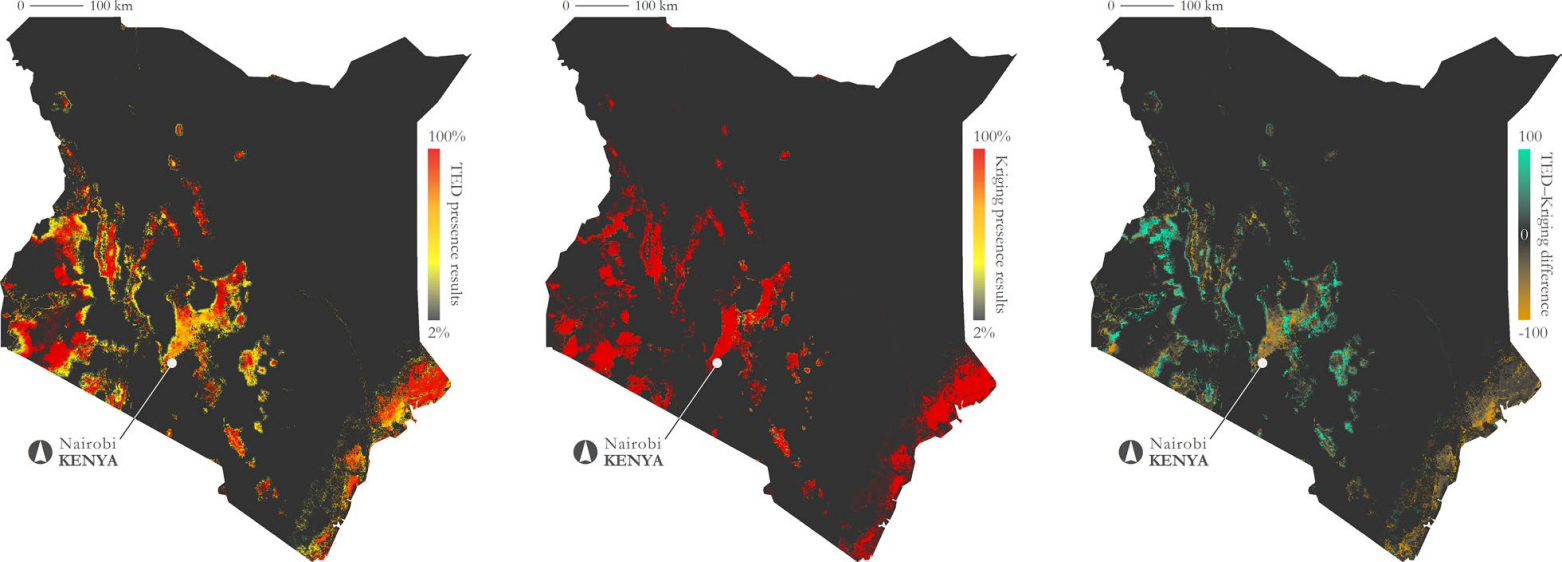
Comparing tsetse presence frequency of the GEE-TED and kriging models (2016–2017). Left: GEE-TED model predicted presence probability; middle: kriging model predicted presence probability; right: differences in results (kriging probability subtracted from GEE-TED probability)

Model accuracy was also characterized across the complete temporal range (2016–2017). Figure 9 illustrates the frequency of inaccurate predictions for absence, presence, and both. Overall, prediction inaccuracy on average for both presence and absence was 25.2%, though some areas are consistently predicted inaccurately (e.g., western Kenya near the city of Kitale). The average inaccuracy of presence only was 26.8% and the average inaccuracy of absence only was 21.7%. Figure 10 shows the frequency of predicted presence across the complete temporal range for both the GEE-TED and kriging-based models. The second map in the Fig. 10 series demonstrates the stability of the kriging approach and the third map highlights the differences between the two models.

## Discussion and conclusions

### Tsetse control and nagana eradication

The discourse around eradication versus control of tsetse has evolved from total eradication to mitigation to management strategies. Ontological uncertainty remains. Our position is that tsetse control and disease management are most effective in places where tsetse persist in small numbers or only for short time periods. Refugia management, whether through the strategic placement of targets that reduce populations or prevent expansion of the flies into new areas, elimination of a substantial portion of the female tsetse population, or the intensive synoptic spraying of insecticides, tend to be short-lived or spatially constrained solutions. Many East African national parks use the encircling targets method both to keep tsetse from invading nearby pasture lands but also to keep pastoralists with trypanosomiasis susceptible cattle out of the parks. The purpose of the original TED model and the revised GEE-TED model was the identification of tsetse refugia. In some corners, management or eradication of refugia is viewed as a viable solution. However, disease management, which should arguably be the goal, will allow the alternative of control. Seasonally, tsetse areal coverage expands and contracts with the rainfall, suitable soil moisture, and presence of food sources. It is in these areas that a rapidly growing population of tsetse meet with the transitory food source of cattle. The cattle herds travel long distances and are the obvious conduit for nagana and trypanosomiasis spread. By minimizing the opportunities for these two distinct groups to meet, disease mitigation efforts are maximized. This project sought to offer an alternative to the deterministic TED/GEE-TED model that produces a 45 day post-data collection map of tsetse presence with a geostatistical solution that presents the probability of presence across all spaces and times.

The BME model is a strong alternative to predict tsetse distributions especially given the lack of spatially accurate rainfall forecasting and delayed processing of remotely sensed data collectively in the − 45 days to present to 180 days in the future temporal window. In previous work, we explored the links between climate and tsetse [77] and demonstrated the value of temperature predictions and unreliability of the rainfall predictions. In future studies, incorporating climate change model data into the BME modeling environment could easily be accomplished. The mean trend of the BME model in this analysis is constructed by using the average tsetse presence for each space–time period which is partially a function of climate. The mean trend indicator could easily be replaced in future studies by a climate change model to enhance the model’s prediction abilities. This new mean trend could be a linear regression model that incorporates the effect average temperature, rainfall and vegetation changes would have on each location’s tsetse presence [78]. Various studies have used linear regression and other models to replace the mean trend to enhance the prior dataset of the BME model [79–82]. BME approximates a normal distribution, but the errors of omission explicitly minimized with GEE-TED and TED are less likely to be equally represented across both methods. With BME errors of commission are less likely as well, albeit still present as the commission errors embedded within the GEE-TED model are retained.

### Parameterization and-use/land-cover

GEE-TED relies on parameterization to produce reliable results. Before using the model, it is critical to understand the temperature, available moisture, and LULC suitability requirements for a specific species in a specific geography, e.g., *Morsitans* (savannah) and *Palpalis* (riverine) tsetse groups in Kenya will require different model parameters. This need for species-specific parameterization is expressed in the results from GEE-TED and the BME kriging model. The spatial distribution of *Palpalis* and *Fusca* tsetse groups across Kenya is not conveyed in the results presented here since their behavior and habitats differ from the Morsitans (savannah) tsetse. However, with the framework and code provided, only minimal modifications are needed to retrofit GEE-TED for use with other, or multiple, varieties of tsetse. NDVI used here as a proxy for soil moisture/relative humidity [83] but may not be generalizable across species/geographies and a thorough review will be needed to identify a suitable threshold.

LULC accuracy in Africa is integral to GEE-TED. While the TED model is designed to accommodate timeseries LULC input, GEE-TED currently uses a static composite of MCD12Q1 (versions 051 and 006) due to interannual uncertainty in the classifications [77]. Future versions of GEE-TED will include timeseries LULC as more reliable products are ingested into the GEE repository. One such dataset is the Copernicus Global Land Cover Layers Collection (CGLS-LC100) derived from the PROBA-V satellite beginning in 2015 and delivered annually through 2019 at a 100-m spatial resolution, continuation of which is planned using Sentinel-2 [84]. CGLS-LC100 is presently global; however, the first iteration (Collection 1, 2015) was focused on the African continent and showed an overall accuracy of 74.3 ± 1.8% [85]. The current release (Collection 3, Level 1) is an improved model, showing an overall accuracy in Africa of 80.3 ± 1.9% in 2015. The overall accuracy of Level 2 (2015) is 76.8 ± 2.0% [80]; Level 2 differentiates between open and closed forest types, which is important for delineating suitability for different tsetse species [86]. The fine spatial resolution of CGLS-LC100 (100-m), compared to MODIS (MCD12Q1, 500-m), is likely to prove beneficial in complex landscapes such as Sub-Saharan Africa where LULC mixing takes place at fine spatial scales.

## Conclusions

### Model generalizability and accessibility

These models, and any species distribution model, must have a minimum mapping unit that works with the biology and system dynamics of interest. Here, we are fortunate that the flying distance population leading edge expansion rates match the spatial resolution of the MODIS data product. While it would be trivial to program, albeit non-trivial computationally, the biology of the system does not require finer spatial resolution and in fact would likely introduce false precision in the presentation of the results. While a popular path to publication, improved spatial resolution is not needed here. With tsetse and nagana, high temporal frequency of data collection and rapid processing of those data by the providers is critical. The many papers using static landcover suffer similarly from two primary problems: the obvious limitation of time misses both seasonal effects as well as important patterns emerging from climate change. One notable example of the latter is the expansion of woody brush in North East Kenya, effectively captured both in the time series land cover products and anecdotally through the regional development and tsetse literature.

Among the benefits of model integration in GEE is the flexibility to accommodate continually updating datasets. The MODIS temperature, NDVI, and land-cover datasets are regularly updated and GEE-TED can be run at any time using new date ranges. The model is also generalizable across geographies, accepting any country or region of interest. Kenya is geophysically complex, maintaining stable albeit discontiguous tsetse populations, and suffering a high burden of AAT. Adapting the TED model, previously validated in Kenya, for use in GEE is a major milestone in accessibility given that GEE eliminates the costly software requirements of other GIS platforms and the hardware requirements for processing large volumes of geospatial data. Additionally, GEE-TED is not limited to MODIS products and can be altered as new datasets become available. Four years from now, a full decade of imagery collected by the Sentinel-2 constellation will be available at 10- and 20-m spatial resolutions. We found parallel computing in tandem with Monte Carlo methods granted the ability to analyze significantly larger datasets (more than 2 billion points) at finer spatiotemporal scale than had previously been possible. This methodology is particularly useful for large regulatory or health studies that necessitate a country wide data analysis at a fine spatiotemporal scale; examples include predicting air quality for the continental United States. It is also useful for models that require data from multiple model simulations [49].

Conversely, BME kriging can be used to generate results in a more timely and computationally efficient manner by: (1) utilizing coarser spatial resolution imagery (e.g., 1000-m instead of 250-m), depending on the user’s scope/needs; (2) reducing the sample area to approximately the size of a few United States counties; (3) increasing the temporal scale to months or years instead of approximately every 16 days. Shrinking the spatiotemporal grid size will allow the analysis to be performed on desktop workstation. The GEE-TED script is also capable of both resampling to coarsen the spatial resolution and masking to reduce the output area. Furthermore, with a minimized spatiotemporal scale, the BME analysis can be fully automated, i.e., calculation and selection of the mean trend, covariance, and BME estimation using the freely available BMEGUI software. BMEGUI relieves the necessity for high-level programming skills and results generated through a BMEGUI analysis are indistinguishable from BME analyses performed on other platforms such as MATLAB [59].

Initializing GEE-TED and the BME analysis in Kenya and generalizing it to other countries is relevant to the current study of AAT. In 2020, the European Commission created, “Controlling and progressively Minimizing the Burden of Animal Trypanosomosis” (COMBAT) program [87, 88]. The COMBAT project refers to AAT as a “scourge” for livestock in continental Africa and notes the possibility of its spread to Europe if not properly contained [88]. The program’s goals include a sustained, continent-wide reduction of AAT; an increased understanding of the geographic expansion of AAT; and improvements in focused and efficient disease control [87, 88]. There has been limited progress in AAT control over the past 20 years in contrast to the successful reduction in HAT [87]. The methods described in this article can contribute to the goals of that program.

## Data Availability

GEE-TED uses public domain NASA products: (1) MOD11A2 v006 Land Surface Temperature, (2) MOD13Q1 v006 NDVI, and (3) MCD12Q1 v006 Land Cover Type, all of which are available through LPDAAC repositories (https://lpdaac.usgs.gov/products) and the Earth Engine Data Catalog (https://developers.google.com/earth-engine/datasets). Code for GEE-TED is open access on Harvard Dataverse via Peter and Messina [56] at 10.7910/DVN/6JR87X. The dataset entry also contains a GeoTIFF of tsetse probability across Kenya for the years 2016 and 2017 and an interactive map is available at https://cartoscience.users.earthengine.app/view/gee-ted. Code for BME can be found at: https://mserre.sph.unc.edu/BMElab_web/?_ga=2.63989164.2082491905.1645121087-914710591.1617131408.

## Abbreviations

AAT: African Animal Trypanosomiasis
BME: Bayesian maximum entropy
GDP: Gross Domestic Product
GEE: Google Earth Engine
HAT: Human African Trypanosomiasis
LST: Land surface temperature
LULC: Land-use/land-cover
MSE: Mean squared error
NDVI: Normalized Difference Vegetation Index
S/TRF: Space/time random field
TED: Tsetse ecological distribution
